# Supplementary material for: Effects of Dietary Folic Acid Supplementation on Sex Differences in Oriental River Prawn, Macrobrachium nipponense
Source: Animals (Basel). 2023 Nov 28;13(23):3677. doi: 10.3390/ani13233677 (PMC10705086; doi:10.3390/ani13233677)
Supplement: Supplementary file 1 [file animals-13-03677-s001.zip › animals-2675324-supplementary.pdf]

Table S1 Primers were used for qPCR verification in this study

Target gene	Sequence (F:5'-3')	Sequence (R:5'-3')
HSP90A	TGAACGACGAGACCGAGA	AAGCCGACACCAAACTGA
HSPA1	CCATCACCAACGACAAAG	TTACCGCCCATCAAAAAT
VNN	TGCTGTTCACTTTATCCA	TACTTCACCTTCTGCCTT
ATP5J	ACTTTGGTCTTTTGGTGG	AAGGCTCTGGAAGGATGC
CACNA1B	AGCAAGGAGAATCTGAGT	CAATGGTAAAGAACGAAG
HK	ACGAAGTGCGATTAGGGT	GGGAAGGAAAAGGTGAAG
FASN	CAGGTGCTTATTTGTTCC	CTGCTTTGACTGTTGTGG
ACACA	AGTTCTGGCTCCCCTATT	TTTGATGCCTTCTTTGTA
ACSBG	GGAAGCAGCAACAGTCAC	AGAAGCGCAGGATAGAAA
ALDH	GACTAAAATCACCTCCCC	CTTCTCAGTATCCGACCA
β-actin	TATGCACTTCCTCATGCCATC	AGGAGGCGGCAGTGGTCAT
